# Explaining the challenges and adaptation strategies of nurses in caring for patients with COVID-19: a qualitative study in Iran

**DOI:** 10.1186/s12912-022-00937-8

**Published:** 2022-06-28

**Authors:** Seyed Fahim Irandoost, Javad Yoosefi Lebni, Hossein Safari, Farhad Khorami, Sina Ahmadi, Goli Soofizad, Farbod Ebadi Fard Azar

**Affiliations:** 1 Social Determinants of Health Research Center, Clinical Research Institute, Urmia University of Medical Sciences, Urmia, Iran; 2 Health Education and Promotion, Social Determinants of Health Research Center, Lorestan University of Medical Sciences, Khorramabad, Iran; 3 Health Promotion Research Center, Iran University of Medical Sciences, Tehran, Iran; 4 School of Nursing and Midwifery, Qazvin University of Medical Sciences, Qazvin, Iran; 5 Clinical Psychology, Islamic Azad University, Kermanshah Branch, Kermanshah, Iran; 6 Department of Social Welfare Management, Social Welfare Management Research Centre, University of Social Welfare and Rehabilitation Sciences, Tehran, Iran; 7 School of Public Health and Safety, Shahid Beheshti University of Medical Sciences, Tehran, Iran

**Keywords:** COVID-19, Coronavirus, Challenges, Adaptation strategies, Nurses

## Abstract

**Background:**

Nurses, as the primary human resource in the fight against COVID-19, encounter several obstacles and concerns. As a result, the current study used a qualitative method to describe the problems and adaptation techniques of nurses caring for COVID-19 patients.

**Methods:**

The current study used a qualitative conventional content analysis technique with 30 nurses working in COVID-19 wards in Tehran hospitals. Purposive sampling, snowball sampling, and semi-structured interviews were used to get access to participants and gather data. The data was examined using conventional qualitative content analysis and the MAXQDA-18 program. To assess the quality of study findings, Guba and Lincoln’s trustworthiness criteria were fulfilled.

**Results:**

The data analysis revealed two main categories and sixteen subcategories: (1) experiences and challenges (lack of protective equipment, high work pressure, marginalized physical health, problems related to the use of protective equipment, being excluded, a lack of a supportive work environment, problems related to patients, psychological problems, fear, marginalized personal and family life, and the challenge of communicating with patients’ families); and (2) adaptation strategies for work conditions (performing religious-spiritual activities, creating an empathetic atmosphere in the workplace, spiritualizing their work, trying to convince the family and gaining their support, and strengthening their sense of self-worth and responsibility).

**Conclusion:**

Nurses’ working conditions can be improved by providing adequate protective equipment, a suitable work environment, and more social and financial support; paying more attention to nurses’ physical and mental health; and considering appropriate communication mechanisms for nurses to communicate with their families and patients’ families.

## Background

COVID-19 is a coronavirus-related acute respiratory disease that first appeared in December 2019 in Wuhan, China [1–3]. Although the entire world has experienced infectious diseases in the past, the breakout of COVID-19 as a novel infectious disease has severely disrupted the public health systems of several countries [4, 5]. The infection has spread to the majority of the world’s countries, and by April 26, 2021, the total number of people diagnosed with COVID-19 was 510,099,967, with 6,245,628 fatalities. The United States has the highest COVID-19 mortality rate at 1,018,582. Iran is one of the nations with the greatest number of COVID-19 infections, with 7,217,117 people infected and 140,996 deaths [6].

Therefore, healthcare professionals (physicians, nurses, and other healthcare staff) perform the most crucial role in treating patients when there is an emergence of infectious diseases, and therefore, their health or even lives are threatened. In other words, healthcare personnel, particularly nurses, are at the forefront of all efforts to prevent pandemics. In other words, healthcare personnel, particularly nurses, are at the vanguard of all efforts to avert pandemics [7]. During a pandemic, nurses conduct research, spread policies, and, if required, increase their level of knowledge to promote public health [8]. Although nurses are primarily responsible for direct patient care, they are prepared to respond to pandemics at any level, including advising governments on research, working with public health teams, and developing humanitarian action plans for COVID-19 patients [9]. Nurses in the COVID-19 pandemic have devoted their lives to fulfilling their duty to battle the disease. They are in direct contact with this virus. Although nurses must take care of not only the patients, but also themselves and their families in such a disaster [10]. Nurses must make sure that all patients get personalized, high-quality care, no matter how sick they are [11].

The health condition of healthcare professionals, particularly nurses, who serve as the primary human resource in the defense against this disease, was jeopardized, resulting in the deaths of many of them [12]. In China, 1716 health staff were infected with COVID-19, with five of them dying [13]. In Italy, nurses had the greatest number of infected healthcare professionals [14]. Liu, Yang, et al., 2020 discovered that healthcare workers who treat COVID-19 patients are more likely to suffer from mental health issues such as anxiety, depression, insomnia, and stress [15]. At the time of the outbreak, 44.60% of Chinese nurses and physicians reported symptoms of anxiety (44.60%), depression (50.4%), insomnia (34%), and stress or anxiety (71.5%). In addition, Liu et al., 2020 found that during the COVID-19 outbreak, healthcare providers faced complexities such as feeling responsible for reducing patients’ suffering, working in a completely new environment, burnout due to heavy workload and protective equipment, fear of becoming infected and infecting others, feeling unable to manage patients’ condition, and managing relationships in these stressful situations [16]. During a study of nurses who cared for COVID-19 patients, Sun et al., 2020 found that nurses had negative emotions in the early stages, such as fatigue and helplessness because of hard work, fear, anxiety and worry; to adapt to the situation, they used personal adjustment styles like psychological adjustment and team support [12].

Healthcare professionals are a vital resource for any country. However, their health and safety are important not only for the safe and continued treatment of patients, but also for the containment of any disease outbreaks that might happen. On the other hand, in order to effectively support healthcare providers and nurses and provide a safe environment for them to perform their job duties during critical periods, particularly the COVID-19 outbreak, it is critical to gain new insights by recognizing their experiences and challenges in work environments, as well as their strategies for adapting to this situation. This will be possible through a qualitative approach, and while qualitative research has been conducted around the world to examine the experiences and challenges of health staff and nurses in relation to COVID-19, no study that investigated these experiences from a qualitative perspective has been found in Iran. Qualitative research is ideal for gaining more in-depth information on different situations (such as the situation of nurses during COVID-19), and the data acquired is based on the participant’s views and experiences. This issue contributes significantly to a better understanding of the phenomena under study. Therefore, the current study used a qualitative method to describe the problems and adaptation techniques of nurses caring for COVID-19 patients.

## Methods

### Study design

This research was performed in Tehran, Iran, using a conventional qualitative content analysis method. The researchers employed this research method since the goal of this study is to understand the phenomenon rather than anticipate it and the subject of the study is complicated. In the conventional content analysis, the categories are derived concurrently with the content analysis of the interview text. Through this method, the researcher acquires a better grasp of the desired phenomena. Another benefit of this type of content analysis is that the data is gathered in a simple and clear way, without the need for a predefined category or theory [17].

### Setting and sampling

The study included nurses who worked in COVID-19 wards at Milad, Haft Tir, Yaftabad, Lolagar, Imam Khomeini, and Masih Daneshvari hospitals in Tehran. The nurses who took part in this study worked on the COVID-19 ward, had a bachelor’s or master’s degree, were in good physical health, and were eager to participate. Furthermore, at the time of the interview, they had not been diagnosed with COVID-19 by a physician. Nurses in other wards of the hospital, on the other hand, were either hesitant to be interviewed and participate in the study, or had been diagnosed with COVID-19 and were therefore disqualified from the study. Purposive sampling was utilized initially, followed by snowball sampling, in this investigation. The researcher initially obtained the contact information and addresses of five nurses who worked in the COVID-19 ward through hospital friends, and the remainder of the participants were introduced by these nurses. The researcher talked to the participants on the phone about the purpose of the research and how it was going to be done, and then asked them for the time and place of the interview.

### Data collection

From April 1 to May 4, 2020, data was gathered using semi-structured interviews. The majority of the interviews (20 cases) were performed in person, with the remainder completed over the phone in Persian. Face-to-face interviews were conducted in order to first gain the participant’s trust so that he/she could supply us with better and more comprehensive information, and second, so that the researcher could better comprehend and code the data. The first author of the study, who is a doctor of health education and health promotion and a qualitative research specialist, conducted the majority of the interviews (21 cases). When scheduling the time and location of the interview, the researcher told the female nurses that they could interview the female researcher if they wanted, and only five of them said they wanted to be the female researcher. When female nurses preferred a female interviewer, a qualified researcher with experience in qualitative research was employed. The participants choose the location of the interview. This implies that the location of the interview was questioned after the initial arrangement. Some participants responded that they would like to be interviewed outside of work, while others said they would prefer to be questioned at work. In certain circumstances, individuals would rather be questioned during a break or after working at home. These interviews were held at a time and location when only the participant was present. The nursing department was allowed access to the hospital, but the participants were not chosen by them.

To ensure a comfortable atmosphere, the researcher offered a brief summary of his or her personal and educational background at the start of the interview, then emphasized the importance of the research and how to conduct and report it. Following the receipt of signed consent, the research proceeded with a few demographic questions. The interview questions (Table 1) were created in such a manner that, after studying the sources and consulting with experts (in order to better understand and design questions for the interview, three nursing professors and three nurses who had worked in another hospital where the interview was not conducted were used). All of the authors of the paper initially generated 7 questions for the interview over two sessions of discussion about the research objectives. Then, during three experimental interviews, these seven items were examined to determine if they might lead to the research objectives. In another meeting, after three experiments were done, two more questions were added to the interview questionnaire to finish it off.

**Table 1:** The guide for interview question

No	Questions
**1**	What happened when the first COVID-19 patient was admitted to your ward?
**2**	Are your job duties changing these days as you handle COVID-19 patients? Explain.
**3**	Has your mental or physical health changed since you began treating COVID-19 patients? Explain.
**4**	What are the most difficult issues you confront when caring for COVID-19 patients?
**5**	How do those around you react when they learn you are in contact with a COVID-19 patient? Explain
**6**	Are you happy with the services that your employer offers these days? Explain.
**7**	What is the difference between caring for COVID-19 patients and other patients? Explain.
**8**	How do you deal with this situation better? Explain.
**9**	How do you adjust to this new circumstance (nursing COVID-19 patients)? Explain.

The participants were asked all of the guiding questions. The sequence of the questions was not the same for all of them, and it altered in some cases based on the responses of the participants. Notes were collected as needed during the interviews. The goal of taking notes was to assist in recalling some of the participants’ thoughts, sentiments, and moods for analysis and reporting. It also aids comprehension of the concepts and outcomes. In reality, because qualitative research is done over time, there are a few things and concepts that should be kept in mind.

The average length of an interview was 50 minutes. The interview times were set at 40 and 70 minutes, respectively. Data collection was done with the help of 30 nurses and the theoretical saturation criteria, which is the primary sampling criterion in qualitative research. Indeed, when no new interviews added a new notion to the inquiry, the researchers realized they had reached theoretical saturation [18, 19]. Since the research team reviewed each interview after it was completed, the repetition of a code (finding) throughout the interviews generated saturation on that issue. As a result, saturation and completion of the data collecting procedure were considered when the codes in the interviews were nearly totally duplicated and the interview merely delivered existing data to the researchers rather than providing new information. However, 5 further interviews were undertaken after the codes were repeated for greater assurance [20, 21]. Nothing new was discovered.

### Data analysis

The data was managed using the MAXQDA-2018 program. The first and second authors of the essay analyzed the data. It should be mentioned that the data analysis procedure was communicated to all researchers, and the categories and subcategories were designated after synthesizing all of their perspectives. For data analysis, the Graneheim and Lundman approach, which consists of five phases, was used [22]. In the first phase, the author attentively listened to the interview recordings numerous times and transcribed them word for word. This was done with a sound recorder and an audio player. To ensure that the interviews were correctly transcribed, author 5 listened to the recordings again and rectified any errors in the transcriptions. The researchers studied the text of the interviews multiple times in the second stage to gain a general understanding of the content. The sentences were then carefully examined line by line in the third stage, and the starting codes were recovered. In the fourth phase, the researchers grouped all of the codes that were conceptually and contentally similar into one category. The data was then organized into primary categories that were more generic and conceptual, and the themes were retrieved at the end.

### Ethical considerations

To adhere to ethical issues, the first step was to speak with two expert physicians working in the field of COVID-19 to ensure that the interviews were done in a way that presented no risk to the health of the participant or the researcher. Hence, all health conditions were noticed during the interview procedure. During the interview, health precautions were followed, which included keeping a distance of 2 m between the researcher and the participants. Participants and researchers also wore masks and gloves and disinfected the area with alcohol. Some interviews were also conducted at nurses’ homes or in remote parts of the hospital that were available to the public. All participants signed the study’s informed consent form. In situations where telephone interviews were conducted, the permission form was provided to the participant, who signed it after reading and verifying it and sending a copy to the researchers. In face-to-face interviews, the form was signed by the participant before the interview began. Participants were informed that their names and identities would not be revealed in the results. They were also informed that they were under no obligation to participate in the study and might opt out of the interview at any moment. All interviews were taped with the participants’ permission.

### Trustworthiness

To ensure the data’s integrity and reliability, Guba and Lincoln’s criteria [23] were fulfilled. In addition, 32 elements from the qualitative study report by Tong et al. were highlighted [24]. To establish credibility, items such as selecting participants with the greatest variety, offering comments on the outcomes, and checking the results were completed. Parts of the interview text, as well as all extracted codes, categories, and sub-categories, were checked and approved by 5 observers who were not part of the study team and were familiar with the qualitative technique in order to achieve confirmability (Table 2). Participation and use of all authors’ viewpoints in the analysis and coding were deemed crucial for dependability. When analyzing and coding data in qualitative research, the more authors there are and the more diverse their disciplines, the more desirable the data analysis and coding are and the clearer the different dimensions of the problem are, because each author is familiar with the problem based on his expertise and experience, and together they provide a better analysis of the data [20]. Some items were observed in order to obtain the transferability criterion, such as the frequent use of direct quotations from research participants, a complete description of the research steps and how to complete them, and providing the research results to 5 nurses from the study who had similar conditions as the participants and obtaining their approval.

**Table 2:** Profile of experts familiar with qualitative research

No	Age	Gender	Occupation	Major
1	45	Male	University professor	Nursing
2	38	Male	University professor	Health Education and Promotion
3	47	Female	University professor	Sociology
4	55	Female	University professor	Psychology
5	40	Female	University professor	Nursing

## Results

Table 3 shows the demographic information for the 30 nurses that participated in this study. Following data analysis, 120 key codes, 16 subcategories, and two main categories were found (Table 4), which are included together with quotations and explanations.

**Table 3:** Demographic information of the participants

variables	Group	Frequency (%)
Age	Under 25	6 (20)
	25–40	15 (50)
	Over 40	9 (30)
Gender	Male	12 (40)
	Female	18 (60)
Marital status	Single	10 (33)
	Married	20 (67)
Job experience	Under 5	12 (40)
	5–10	8 (27)
	Over 10	10 (33)

**Table 4:** Codes, subcategories, and categories extracted from data analysis

Categories	Subcategories	Codes
**Experiences and challenges**	Lack of protective equipment	Lack of protective equipment such as gloves, high quality masks, face shields, disinfectant solution, and isolation clothes
	High work pressure	Increasing work shifts, increasing number of patients, more visits to patients due to too many drugs and giving different serums, doing patients’ work due to not having a companion
	Marginalized physical health	Fatigue, headache, muscle fatigue, weakness, lethargy, sleeplessness, low quality of sleep, digestive problems, disrupted eating hours and eating habits, menstruation problems of female nurses
	Problems related to the use of protective equipment	Shortness of breath, facial ulcers, heat, sweating, body burns, itching, and leg wounds
	Being excluded	Relatives run away from dealing with nurses, inappropriate behavior and avoidance of neighbors, inappropriate behavior of others outside the workplace, and inappropriate behavior of family members
	Lack of supportive work environment	Not having a suitable place to rest, crowded break rooms, lack of motivational stimuli such as material rewards, worries about dismissal, lack of psychological counseling to cope with the stress related to COVID-19, and lack of adequate training in how to deal with COVID-19 patients
	Problems related to patients	Getting abused and bullied by patients, patients’ boredom, patient’ homesickness, seeing patients in bad condition
	Psychological problems	Depression, self-morbidities, and grief over losing a colleague due to COVID-19
	Fear	Fear of being infected, fear of carrying and transmitting the virus to their families, the disease’s persistence, and ignorance of the COVID-19 virus
	Marginalized personal and family life	Disconnection with family, homesickness for family, reduction of the role of mother or father in the family, being distant from the family during Nowruz, not attending their child’s birthday party, disruption in life plans such as marriage, cancellation of family travel plans
	The challenge of communication with patients’ families	Difficulty in informing families about positive test results, difficulty in informing families about their patient’s death, difficulty in informing families about their patient’s exacerbated condition, accusing the nurses of being shirkers by the patient’s family, too much contact by the patient’s family
**Adaptation strategies for work conditions**	Performing religious-spiritual activities	Praying, saying daily prayers, listening to the Qur’an, saying blessings to Prophet Muhammad [Salawaat], asking for help from the Imams
	Creating an empathetic atmosphere in the workplace	Creating emotional relationships with patients, strengthening relationships with other colleagues, forgiveness and devotion, helping colleagues, giving positive feedback to colleagues, talking more with colleagues, and providing an atmosphere for jokes and laughter
	Spiritualizing their work	Considering their work as jihad in the way of God, equating death due to COVID-19 with martyrdom, and receiving rewards in the hereafter
	Trying to convince the family and gaining their support	Explaining the necessity of being in the hospital, explaining the importance of nurses’ work, observing health principles and comforting the family, showing working conditions to family members, and making phone and video calls to the family
	Enhancing their sense of self-worth and responsibility	Feeling satisfied with strengthening their social image and status among people, satisfaction with people’s gratitude, increasing their efforts to save people, increasing interest in their work and profession, taking responsibility for people’s health

### Experiences and challenges

The emergence of COVID-19 in Iran, as well as the hospitalization of its victims, has impacted the personal and professional lives of nurses, the most crucial medical workers in the COVID-19 fight. A lack of protective equipment, high work pressure, marginalized physical health, problems with protective equipment use, being excluded from work, a lack of a supportive work environment, problems with patients, psychological problems, fear, marginalized personal and family life, and the challenge of communicating with the families of the patients were all present.

#### Lack of protective equipment

One of the most serious issues that nurses had when dealing with COVID-19 illness was a shortage of protective equipment such as gloves, high-quality masks, face shields, disinfection solutions, and isolation garments. This was a source of concern for many nurses, and it jeopardized their health.

*“We didn't have PPE from the start. We had to make contact with people who were suspected of having COVID-19 without PPE on several occasions. The situation has improved recently, but we still need to make significant savings from time to time.”* (p. 4)

*“Two pairs of gloves a day was insufficient for us.”* (p. 6)

*“At first, when there was a scarcity, they provided us with a plain mask that was of extremely poor quality.* (p. 11)

*“In the beginning, we had few hygiene supplies, we had to save a lot of money, and our lives were at risk.”* (p. 3)

In reality, nurses were in the vanguard of the fight against COVID-19, and for this mission to be successful, they needed to have decent working conditions at the outset. However, because of a lack of protective equipment, their working circumstances were hazardous, and the COVID-19 may have been caught at any time.

#### High work pressure

With the increasing incidence of COVID-19, the number of hospitalized patients grew day by day, and many hospitals had a nurse shortage, requiring nurses to work longer hours. Furthermore, because COVID-19 patients had no companions, nurses performed the majority of their work, and due to their physical condition, nurses had to evaluate them more frequently than other patients. Nurses are under a lot of stress at work as a result of these challenges.

*“We’ve been quite busy. The number of patients is growing, and we must work more.” (p. 23) “The COVID-19 patient has a lot more work ahead of him. We have to keep an eye on them and keep an eye on them all the time since they don't have any buddies.”* (p. 2)

*“Because the number of our employees has reduced and the number of patients has grown, we must work considerably harder than before.”* (p.17)

*“We have a lot of work, we don't have a break, I’m becoming weary, I’m getting less”.* (p. 4)

The prevalence of COVID-19 has altered the working circumstances of nurses in a variety of ways. In addition to working longer hours, nurses had to do more work and deal with uncertainty caused by changes in the duty shifts.

#### Marginalized physical health

Nurses’ health suffered as a result of the increased work pressure and psychological issues that developed in certain situations, and they experienced symptoms such as weariness, headaches, muscular exhaustion, weakness, and lethargy. In addition, owing to high job pressure and stress, their sleep quality was poor, and they had stomach issues, interrupted meal hours, and poor eating habits in terms of nutrition. Most female nurses also said that they had a lot of problems during their periods because they did not get enough sleep.

*“There are times when I’m so exhausted that I fall asleep standing up.”* (p. 19)

*“I don't get enough rest and sleep; thus I have a headache; I have discomfort throughout my body.”* (p. 27)

*“Sometimes we become so busy that we can't even eat lunch or supper; recently, my stomach has been bothering me.”* (p. 16)

*“I had menstruation last week, and I was on the verge of dying. I became irritated; I had never been affected by menstruation in my life.”* (p. 5)

As a result of the increasing working pressure and psychological stress caused by the rise of COVID-19, most nurses’ physical health has been jeopardized, and if these conditions persist, they may face more serious problems.

#### Problems related to the use of protective equipment

The majority of the nurses reported that using protective equipment was difficult for them and caused them several problems, such as face ulcers, shortness of breath, heat, sweating, body burns, itching, and leg sores. Due to the spread of COVID-19, nurses were required to pay greater attention to health concerns and work for long periods of time while wearing protective equipment to prevent contracting the disease. This made their job much more difficult.

*“When I wear isolation clothes, I get very hot, so I sweat a lot and my skin burns.”* (p. 12)

*“Since I have been wearing these clothes, my skin has become sensitive and it itches all the time. I am very bothered.”* (p. 14)

*“I get short of breath when I use shields, masks, etc. I often feel suffocated.”*(p. 21)

Nurses had to wear protective equipment such as masks, face shields, and insulated clothing for a long period due to infections in the hospital environment and an increase in working hours, which caused numerous issues for them.

#### Being excluded

Most nurses reported being neglected by friends, neighbors, and even family members outside of the hospital, and many individuals discontinued contact with them. Because COVID-19 may be spread in a variety of ways, nurses who come into contact with patients are at a higher risk of contracting the disease. People are terrified of nurses as a result of this. Furthermore, since the disease can spread without symptoms, people are more afraid of nurses, which leads to them being excluded from society and even from their own families.

*“Most of our family members no longer greet me cordially. They are afraid I will go to their residence. I ran across them numerous times outside, but they never said hi.* (p. 26)

*“Our neighbor's wife urged my mother to tell her daughter not to return home and stay in the hospital, or we, the inhabitants of the building, will have to evict you.’”* (p. 4)

*“My brother-in-law no longer allows my sister and her children to visit us because he is afraid I will take COVID-19. I am quite irritated by their actions.”* (p. 8)

*“When people discover that we are nurses; they treat us terribly. I once grabbed a cab and told the driver I needed to go to the hospital. When he discovered I was a nurse working in the COVID-19 unit, he refused to drive me there and forced me to get out of the car. For a few days, his conduct concerned me.”* (p. 29)

*“My husband frequently advises me to quit working; he says, ‘you’ll take COVID-19, and we’ll be sad, but I can’t; my conscience won’t let me.”* (P. 14)

*“One of the most difficult issues for us, nurses working in the COVID-19 ward, is that many of our families do not support us when we most need it. My friend's father allegedly told her, “I’ll pay you twice the money the hospital provides you if you don't go to work.”* (p. 20)

#### Lack of a supportive work environment

With the increase in the number of patients with QOVID-19 and their hospitalization, most hospitals accepted more patients, and in the meantime, nurses had to stay in the hospital for long working hours and endure a lot of pressure, so they needed more attention and help. A portion of this assistance was pecuniary in nature, but the most essential aspect was to offer a secure and healthy atmosphere for them so that they could relax more. However, the majority of nurses were disappointed with the hospital’s service quality. Most nurses were not happy with the quality of service at the hospital, citing workplace issues like a lack of a place to rest, crowded break rooms, a lack of motivational stimuli like money, fears about being fired, and not enough training on how to deal with COVID-19 patients.

*“We all have to stay at the hospital, but there isn’t anywhere for us to relax. It can become too crowded at times, making it impossible to take a break.”* (p. 9)

*“The officials constantly laud us but do nothing to make us happier or more hopeful. They didn't provide us with any tangible prizes.”* (p. 3)

*“Since the arrival of COVID-19, we have experienced the most tension and worry. Our days and nights have been messed up, but no counseling has been offered to help us relax.” (*p. 12*)*

*“I was perplexed during the early stages of COVID-19’s proliferation. I did not know exactly how to treat patients. I was afraid of them because we weren’t trained for such a situation. ”*(p. 28)

#### Problems related to patients

COVID-19 patients’ circumstances differed from those of other hospital patients. They couldn’t get emotional support since caregivers were not allowed to be there, so they were under additional strain. A number of nurses reported being assaulted and bullied by COVID-19 patients. They were particularly disturbed to observe COVID-19 patients in bad health. Furthermore, because COVID-19 patients were alone, they felt lonely and homesick at the hospital, making it difficult for them to cope with the hospital circumstances and, in some cases, causing them to have conflict with the nurses.

*“Patients who had been in the hospital for a few days grew quite homesick and impatient, and we all had to calm them down.”* (p. 19)

*“Some patients were rude to us and insulted us.”* (p. 7)

*“Some COVID-19 patients were sick of being hospitalized. They were less tolerant, and they occasionally battled with our coworkers.”* (p. 6).

#### Psychological problems

The COVID-19 outbreak had made nurses’ working conditions extremely difficult since, on the one hand, they were under pressure at work and had some type of hypochondriasis. On the other hand, they had to deal with the sickness and death of their coworkers on a daily basis. The majority of nurses reported feeling depressed as a result of the complicated and demanding situations that had evolved. Furthermore, several of them had self-morbidities and were certain they had COVID-19. Another psychological issue that nurses faced was the sadness of losing colleagues. Some of them were outraged and concerned that they might witness the death of a coworker or that they would be forced to stay at home.

*“I was under the illusion that I had become infected for a brief period of time. I tested negative both times. I even instructed my family on what to do if something were to happen to me.”* (P. 1)

*“I used to be stronger, but now I'm losing patience and tolerance, and I'm constantly thinking about bad things. I think I've become gloomy, and I'm quite impatient.”* (p. 18)

*“I’m always under the impression that I'm unwell. I was really cautious, yet I still believe I am unwell. I always check myself. When I get a cough, I am concerned.”* (p. 23)

*“One of our coworkers died a few days ago. We were furious. My mind is preoccupied with it.”* (p. 6)

*“I am quite disturbed to find that some of my colleagues have been infected and are suffering.”* (p. 17)

#### Fear

Unawareness of COVID-19 transmission and treatment methods had caused fear in nurses, and because they were constantly exposed to COVID-19 (constant exposure to COVID-19 patients) and lacked adequate access to protective equipment, they were very concerned that they would transmit the virus to their families when they returned home. Worry and dread, according to the majority of participants, occasionally impair their lives. In most cases, they were worried about being infected or having the virus, and passing it on to their families. They also were worried about the disease’s long-term effects, as well as the COVID-19 virus.

*“All of my concerns are that I will pass the sickness on to my family, because my father got a kidney transplant and lives with us.”* (p. 11)

*“I’m terrified about contracting COVID-19. I am more scared when I observe the state of the patients, but I don't show it. I sometimes urge my patients not to be scared of COVID-19, despite the fact that I am terrified of it myself.”* (p. 27)

*“I’m concerned that this sickness will spread. Under these circumstances, life has become really challenging for us nurses. I believe we won't be able to bear it if it continues.”* (p. 16)

*“The fact that they discover something new about this disease every day makes me even more afraid since I am convinced that scientists know so little about it and, therefore, cannot find a treatment for it.”* (p. 2)

#### Marginalized personal and family life

As the number of COVID-19 patients in Iran grew, as did the medical staff’s illness and need for rest, hospitals became understaffed, and the remaining nurses were forced to spend more time in the hospital. Therefore, they were no longer available to the family. However, because of the risk of being a virus carrier, many nurses decided to stay at the hospital even when they were off-duty and did not return home, further isolating them from their families. It happened to a lot of other nurses who had to work full-time at the hospital. They did not get married or have their kids’ birthdays.

*“It sometimes takes me more than a week to see my family.”* (p. 8)

*“I haven't seen my 6-month-old baby in a long time; I've missed him.”* (p. 15)

*I was unable to attend my son’s birthday party. I was furious. I was gone from them at a time when my family needed me the most. I don't think I’m a good parent for them.”* (p. 18)

*“My spouse and I were meant to marry in April, but I was always at work, so we couldn’t have a celebration.”* (p. 30)

#### The challenge of communicating with patients’ families

Because the families of COVID-19 patients were unable to accompany them, they were continuously in communication with the nurses to learn about their patients’ conditions, and the nurses were occasionally obliged to relay unpleasant news to the families, such as the patient’s death or deterioration of condition. Most nurses expressed worry about this, citing several issues with patients’ relatives. Also, because the relatives were far away from their patient and couldn’t examine his health carefully when they learned of his death, they frequently accused the nurses of not treating him correctly. This created problems between families and nurses as well.

*“I was irritated at times when I had to inform the patient's family that the result was positive.” I didn't know how to tell them; everyone was terrified of this sickness.” (*p. 3)

*“So far, the patient’s death has been announced to the family three times. It’s an arduous job, and we’re ashamed that we couldn't help them.”* (p.10)

*“When we inform the relatives of a patient’s death, some of them accuse us of being shirkers, and occasionally they even attack us.”* (p. 25)

*“Some families discovered our phone number and dialed it. They phoned so frequently at times that we were anxious and frustrated; they wanted to know about their patient’s status all of the time.”* (p. 11)

*“Some of the people who phoned us or who we called were disturbing us; they expected us to explain the entire treatment procedure to them, and they bombarded us with questions. We had no choice but to switch off our phones at times.”* (p. 22)

### Strategies for adapting to working conditions

As a result of the current situation, nurses took some steps to increase their tolerance threshold or adapt better to new situations, such as participating in religious-spiritual activities. They also tried to make the workplace more empathetic, spiritualize their work, try to get the support of their families, and strengthen their sense of self-worth and responsibility.

#### Performing religious-spiritual activities

People usually turn to religious rituals to achieve peace and quiet in times of calamity and other unexpected and terrifying situations [25, 26]. The COVID-19 outbreak was unexpected, and no one knew when the pandemic would end or whether there would be any way to avoid or treat it. This was seen more in nurses than in others because they were more exposed to death and turned to religious rituals to cope. Some nurses claimed that they relied on religious and spiritual activities such as praying, performing daily prayers, and listening to the Qur’an to lift their spirits and calm themselves. In fact, they attempted to achieve more serenity by approaching God.

*“When I am really weary, I try to say my prayers at an appropriate time; this helps me get rid of sleepiness.”* (p. 28)

*“When I am under a lot of stress, I take advantage of the opportunity to listen to the Quran. Then I feel quite relaxed; it gives me a pleasant feeling.”* (P. 9)

*“When I talk to God and feel that he hears what I’m saying, I relax. I beg him to protect us from this sickness.”* (p. 12)

#### Creating an empathetic atmosphere in the workplace

Most nurses indicated that during the COVID-19 pandemic, their professional and emotional connections at work improved, and they loved and helped each other more than previously. In fact, nurses can make the workplace more pleasant by developing emotional relationships with patients, strengthening relationships with coworkers, forgiving and devoting, assisting colleagues, providing positive feedback to colleagues, talking more with colleagues, and providing an environment conducive to jokes and laughter.

*“I make an effort to communicate with patients more frequently. To be honest, both they and I have calmed down. I occasionally listen to their anguish; they chat about their history and their recollections.”* (p. 14)

*“Since the COVID-19 patients were admitted to our ward, the colleagues have been more helpful to one another since they realized we're all in a terrible circumstance.”* (p. 9)

*“It makes me happy to see my coworkers assisting me. I've only recently begun working. I feel wonderful when they support me.”* (p. 16)

*“When we have more time, we sit and talk with our coworkers. We all like one another and understand each other well, so when we chat to each other, we feel really peaceful. ”* (p. 24)

*“I sometimes make jokes with patients to get a reaction from them, and they like it as well. When I'm not present, they question my coworkers about me. I like to crack jokes with my coworkers because it helps me feel less worried.”* (p. 17)

*“When I’m under a lot of strain, I talk to my coworkers and we switch shifts. They didn’t take it easy at first, but now everyone understands each other, and the hospital has developed a very positive culture. I want it to always be the same, even when the condition is under control. ”* (P. 5)

#### Spiritualizing their work

To deal with the new circumstances, some nurses attempted to spiritualize their profession by viewing it as jihad in the cause of God, associating death due to COVID-19 with martyrdom and gaining benefits in the hereafter. Some nurses considered themselves Mujahideen for the love of God, believing that if they were infected with COVID-19 and died as a result, they would go to paradise since they were killed in God’s name. They also thought that if they died, they would get a large recompense from God in the hereafter.

*“I now believe I am on the battlefield. I'm not scared of death. I used to be terrified, but not now since I'm confident that if I die, I'll go to paradise.”* (p. 11)

*“Our job is nearly like jihad in the name of God. Even if they are paid well, few people will choose to work in these conditions.”* (p. 8)

*“When I consider that I am battling for God and that if I die, I will be a martyr, my bravery grows and I want to work more.”* (p. 26)

*“Despite the fact that I work long hours, I never feel exhausted because I know that God sees everything and knows about my problems, and I am confident that one day he will reward me for my efforts, whether in this world or the next.”* (p. 14)

#### Trying to convince the family and gaining their support

Some nurses indicated that they attempted to get the family’s support in order to obtain assistance from them as a powerful emotional resource. To get the family’s support, they took steps like explaining the value of nurses’ jobs, respecting health rules and calming the family, displaying working conditions to family members, and making phone and video calls to the family to show them what it was like to be a nurse.

*“It was really essential to me that my family was behind me, so I talked to them right away and informed them that if we nurse didn't, the situation would worsen and everyone would become sick.”* (p. 13)

*“I told my family that I would take as much care of myself as possible to avoid becoming infected. I occasionally make a video call with the clothing I'm wearing to show them that I respect them and help them feel better.”* (p. 3)

*“I spoke with my wife and described the working circumstances to her. Thank God, my wife is rational. She accepted the circumstances and was really supportive of me throughout this time, in contrast to my other coworkers, who frequently had issues with their families.”* (p. 18)

*“I try to contact or chat with my parents at least once a day, so they can relax and support me more when they are less frightened.”* (p. 21)

#### Strengthening sense of self-worth and responsibility

People’s and the media’s attention and support for medical professionals, particularly nurses, in cyberspace made nurses happy and contented, strengthening their sense of self-worth and duty.

*“It makes me happy to know that everyone praises us and thinks about us.”* (p. 1)

*“When I see everyone supporting us nurses, I feel that my work is really valuable; I like my work even more than before.”* (p. 27)

*“Previously, I did not enjoy my job. I took the entrance test to study medicine last year, but now I realize how vital my work is.”* (p.15)

*“I used to fabricate excuses and avoid going to work. When I first arrived, I was bored and just thinking about when my work would stop, but this is no longer the case. My sense of accountability for my job has grown.”* (p. 13)

*“I try to focus on the good parts of my work and how useful it is; this makes me feel better about it and makes it simpler for me to work.”* (p. 6)

## Discussion

The current study used the qualitative method to investigate the challenges and adaptation strategies of nurses caring for COVID-19 patients. Their strategies included spiritualizing their work, going to church, setting a good example for others in the workplace and reinforcing their sense of self-worth.

In the current study, one of the main barriers for nurses was a lack of safety equipment. The COVID-19 pandemic was beyond the capabilities of many countries’ health systems. One of the major concerns was a lack of preventative and protective equipment. Previous research has indicated that nurses face a paucity of protective equipment in many parts of the world, in addition to coping with COVID-19 [13, 14, 27, 28]. Because COVID-19 is transmissible and protective equipment is useful, nurses may be unable to perform their duties properly if these materials are not accessible. This problem has also been reported during MERS, Ebola, SARS, and influenza outbreaks [29–32]. During pandemics, there is a lot of demand for hygiene supplies, and governments are not ready for them. This makes it hard for people to get these products.

Other challenges mentioned by nurses in this study include extreme work pressure and a large number of tasks as a consequence of many shift changes, a growing number of patients, more visitors to patients, and performing patients’ tasks since they do not have a companion. Several studies on the difficulties that nurses face during pandemics have yielded similar results [15, 33–35]. It is worth emphasizing in this context that dealing with infectious diseases throws additional pressures and challenges on medical workers. Hospitalization of patients and the lack of a definitive and effective drug for COVID-19 put the most strain on nurses because they have to keep an eye on the needs and physical condition of patients, and patients need physical and psychological help from nurses.

Another barrier for nurses was difficulties with the use of protective equipment, which is consistent with prior research [36, 37]. Fernandez et al., 2020 found that repeated wearing and removal of protective clothing and equipment causes psychological stress in nurses, as well as pain and exhaustion [38]. In fact, nurses had to wear specialized attire for extended periods of time in order to maintain their health, which created a number of limitations and obstacles for them. Furthermore, due to the rapid spread of COVID-19 and nations’ lack of preparedness to battle the pandemic, the equipment developed lacked the necessary standards, and its use disrupted nurses’ normal functioning [39, 40]. In this way, and in line with the findings of this study, previous research found that nurses had a hard time wearing protective gear for long periods of time during the H1N1 flu pandemic [41].

Another study found that nurses’ physical health was marginalized during the care of COVID-19 patients, with symptoms such as tiredness, headaches, muscular exhaustion, weakness, lethargy, sleeplessness, and digestive troubles, which corresponded to the final two concerns (above) of nurses. During a pandemic, workload can lead to a range of health problems, including exhaustion, insomnia, headaches, and anorexia [42].

According to Liu and Aungsuroch, 2019, nurses did not drink water after donning protective equipment in order to avoid going to the restroom and keep the garments off. Thus, working longer hours causes stress, physical and mental exhaustion, and, eventually, job burnout [43]. While relaxation, a healthy diet, and recognizing healthcare professionals’ basic needs are all beneficial to their health, so are health protocols and the use of protective equipment [44]. High job pressure, weariness, and physical conditions generated by patient care, as well as physical obstacles caused by the use of protective equipment for a lengthy period of time, all contribute to the weakened immune systems of nurses, marginalizing their health. The healthcare system will face a variety of difficulties in the future as a major section of its workforce ages and burns out. Previous research shows that nurses face a lot of physical and mental challenges during pandemics [37–40, 45].

According to the participants, nurses spend long hours in hospitals and medical centers due to high job pressure, yet these work environments do not provide them with any material, counseling, or training help. However, studies have shown that organizational assistance, particularly the provision of expert psychiatric counseling in these stressful work environments, may be extremely beneficial and even decisive in decreasing stress and anxiety and affecting sleep quality in medical workers [36]. Given the harsh circumstances of COVID-19, as well as its rapid transmissibility and high death rate, nurses faced a number of challenges and pressures, necessitating the application of psychological skills to reduce the disease’s burden. Meanwhile, a previous study has highlighted a lack of skills and support in the workplace and society as one of the most serious concerns confronting nurses [37]. In line with these findings, a new study found that nurses who did not get enough help were less effective and healthier.

Being shunned by relatives, friends, neighbors, and even family members was another concern raised by nurses. Nurses’ work status in society is met with mixed criticism. Despite societal praise and thanks for nurses’ efforts, labor, and services, they have been accompanied by a type of rejection and stigma, and many have avoided contacting and bonding with them, and even their families, for fear of becoming infected with COVID-19 [39]. Kim, 2018 discovered that during the MERS pandemic, South Koreans mistook nurses for virus carriers and avoided them [31]. Nurses, on the other hand, must keep in touch with family and friends in order to gain spiritual support [46]. Support from family and friends, as well as empathy, are crucial in improving the health and performance of medical staff and nurses [36]. Regarding the exceedingly sensitive state of nurses caring for COVID-19 patients, their seclusion from others and loss of social support add psychological concerns to their professional stress and exacerbate their problems.

Patient-related challenges were another hurdle for the nurses in the current research. Nurses may be mistreated and tormented by patients in this respect, or watching patients’ suffering and concerns, as well as their horrible state, may negatively affect them. In a study conducted by Liu et al. 2020, nurses expressed remorse at the sudden loss of patients’ lives as well as a severe inability to alleviate patients’ pain [16]. According to Marjanovic et al. 2007, one of the nurses’ concerns is the difficulty of saving patients due to the disease’s uncertain nature and lack of clear therapy [47]. Nurses often help people maintain and recover from illness or injury. However, the current spread of COVID-19 has led to a wave of deaths, and the exhaustion and frustration caused by not being able to save patients hurt nurses both physically and mentally.

Another barrier for nurses in the present study was psychological concerns, which might be attributed to causes such as fear of catching the disease, high work pressure, being apart from family, and being unable to save patients and see their deaths. The bulk of the pandemic studies have discovered nurses with mental health problems [42, 48–53]. Yu et al., 2020 reported a substantial frequency of anxiety among nurses as a result of COVID-19 [54]. Lai et al., 2020 discovered that during the COVID-19 pandemic, the majority of nurses in Chinese hospitals reported indicators of mental illness, which is similar to the current study. Therefore, nearly 70 and 50% of nurses, respectively, had experienced anxiety and despair [55]. During the COVID-19 period, nurses have a lot of psychological problems because of the effects and consequences of this disease, and they need extra help.

Another hurdle for nurses in the present study was their worry of getting COVID-19 and passing it on to family members, as well as their anxiety about the virus and its persistence. This fear is justified; as healthcare workers have been linked to more than 30% of SARS deaths [56]. Numerous studies that corroborate our findings mentioned challenges such as nurses’ fear of contracting the infection and spreading it to family members [41, 57–59], fear and helplessness due to disease outbreaks [60, 61], and rapid changes in recommendations and knowledge related to emerging and unknown diseases [62–64]. According to the findings of the study by Cui et al., 2020 the fear of infecting family members was the most important predictor of anxiety and stress in nurses working in the COVID-19 ward [65]. According to research on nurses’ experiences in previous pandemics caused by other types of coronaviruses, such as severe acute respiratory syndrome and Middle East respiratory syndrome, nurses were extremely concerned about infecting themselves and their families. They developed considerable levels of psychological dysfunction symptoms such as stress, worry, and even depression as a result of the condition’s risk of illness and social stress [60–62, 64, 65]. COVID-19 has caused substantial anxiety among nurses because of its enormous unknown and the means by which it is spread, to the point that most nurses are concerned that they are risking the health of their family members as well as their own.

One of the surprising findings was the underappreciation of personal and family life, particularly among married nurses. Nurses encounter a number of problems during the COVID-19 pandemic, including homesickness for family members and the demands of being a wife and mother. Quarantine conditions, as well as the closure of schools and kindergartens, intensify these needs. The onset of this pandemic in Iran, on the other hand, coincided with the ancient New Year festival of Nowruz, during which the long-term presence of nurses at work devalued even little festivities with family members and kept nurses away from personal life. Given that one method of preventing COVID-19 transmission was to reduce face-to-face contact and break the chain of interpersonal relationships, nurses were expected to pay more attention to this issue than other members of the community due to their direct contact with COVID-19 patients, and in most cases, they were even forbidden to communicate with family members. Choosing work over family made them rethink their decision. This made them more tired and stressed, and made them even more tired and stressed.

One of the study’s unique and surprising results was the difficulties in communicating with patients’ families, such as telling them of an exacerbation of the patient’s sickness or the patient’s death. Nurses were distressed to see patients in agony, and they were much more affected when they realized they couldn’t assist them. Because of COVID-19’s attributes, nurses were unable to care for patients using traditional nursing concepts and were compelled to communicate with patients through masks and protective clothing. In this scenario, sympathizing with and cheering up the patients is insufficient; patients in critical condition who were also refused visits from family and loved ones need further condolences from nurses, which was not possible under these conditions. This situation, particularly after the patient died, led the nurses to have conflicting and unexpected feelings, resulting in mental disorders. As a result of the patient’s lack of communication, the patient’s family experienced anxiety, confusion, and, at times, resentment. The main challenges for nurses were treating these disorders and developing innovative treatments. Some families, on the other hand, accused the nurses of shirking because they hadn’t seen how they treated and cared for the patients. This caused a rift between them and the nurses.

Another noteworthy and unusual conclusion in this study was that nurses used creative approaches to overcome hurdles and minimize problems. Engaging in religious and spiritual activities such as praying, reading the Qur’an, and praying every day was one of these strategies. This may reflect a level of fatalism among nurses, which is a psychological reaction that arises when they feel helpless. Another noteworthy and unusual conclusion in this study was that nurses used creative approaches to overcome hurdles and minimize problems. Engaging in religious and spiritual activities such as praying, reading the Qur’an, and praying every day was one of these strategies. This may reflect a level of fatalism among nurses, which is a psychological reaction that arises when they feel helpless.

Spiritualizing their job was another intriguing tactic used by nurses in this study to adjust to their new employment surroundings, and it is similar to the previous strategy. In other words, nursing was regarded as a sacred vocation with a reward in the afterlife, rather than merely a labor. Spiritualizing the profession promotes nurses’ job happiness, their ability to cope with professional stress, and protects them from burnout [66]. In other studies, the issue of “conscience” has been recognized as the most potent incentive for role-playing [67], elevating the nursing profession from a normal job to a sacred vocation [68]. Giving their work a spiritual and specific place improves duty satisfaction and assists nurses in adapting to changing conditions. In many cases, spirituality has been a critical component in fulfilling professional obligations, and many believe that if they devote their lives to helping patients, they will be rewarded in another world, a belief that is especially prevalent in religious nations such as Iran.

Creating an empathetic work atmosphere was another strategy used by the nurses in this study, which is confirmed by previous research; when epidemics occur, nurses demonstrate more active compassion and stronger teamwork than ever before [29, 31]. The conditions that nurses faced during the COVID-19 pandemic can be comparable to those seen on a battlefield, when empathy and mutual support are essential. Hence, there is a better sense of trust, empathy, and collaboration in these settings [69].

In reaction to an outbreak, nurses’ cognitive thinking might adapt. They adjust their cognitive appraisal based on their professional experience on a frequent basis in order to maintain mental balance, lead with compassion, and seek team assistance [70]. It may be possible to control nurses’ fears about the pandemic by working well together in a trust and respect setting [38]. This highlights the need to focus on their psychological health. In hard situations, having good psychological reasoning and the ability to adapt to hard places could help nurses better care for patients and lessen their own psychological and social harm.

Another strategy that helped nurses adjust to the harsh conditions of COVID-19 outbreaks was to boost their sense of self-worth and duty. During a pandemic, professional ethics develop a sense of responsibility. Encouraging nurses to take an active role in anti-epidemic efforts strengthens their professional identity and pride [63]. Previous studies, on the other hand, have demonstrated that nurses’ psychological development during epidemics is impaired [29, 71]. Simultaneously, in the current study, increasing responsibility was useful in helping nurses meet their work commitments. In China, the tagline “Everyone is answerable for their country’s growth or doom” is apparent, heightening nurses’ sense of responsibility during the difficult years of COVID-19 [16]. There was a study done by Fernandez et al., in 2020 that found that even though nurses felt scared and vulnerable, they had a strong sense of duty and responsibility toward patients and felt obligated to serve [38].

The last approach used by the nurses in the current study was to try to persuade and get family support. According to Mo et al., 2020, social support is an important protective factor for psychological resilience since it reduces mental stress and alleviates psychological problems [46]. The support of peers, coworkers, family, and friends can assist people in maintaining emotional stability in the face of risk and stressful conditions [72]. The most important purpose of social support is to serve as a protective agent, lowering or balancing the psychological harm caused by stressful events and ongoing life challenges [73]. Social support can also assist in alleviating the detrimental health impacts of job stress [46]. Given the prevalence of the COVID-19 pandemic and despite multiple risk factors, social support for nurses can improve their psychological resilience [74]. Nurses must maintain contact with family and friends in order to get social and spiritual support. This enables them to fulfill their professional obligations while simultaneously maintaining social acceptance and avoiding alienation.

### Limitations and strengths

This study is one of the few that investigates nurses’ challenges as well as their adaptation strategies in caring for COVID-19 patients from their own perspective, which can provide effective information to hospital administrators so that they can take steps to reduce their problems and allow nurses to work in better conditions. This study also had some limitations, including difficulty in scheduling the interviews. It was one of the main limitations because nurses had difficulty determining the time of the interview due to a lot of work shifts and a lack of adequate rest, and the interview time changed several times. Maintaining health criteria for the interview, such as keeping a safe distance and wearing a mask, resulted in some respondents’ voices being too low throughout the recording, and the researchers asked the participants to speak louder. Another limitation was that several of the female participants preferred female interviewers in order to more easily communicate their issues to the researcher. Therefore, the researchers hired a qualified female researcher with experience in interviewing and qualitative research principles. Another problem with the study was that some people were willing to talk on the phone.

## Conclusion

The findings revealed that nurses who are assigned to care for COVID-19 patients face multiple and multifaceted challenges such as work pressure, a lack of protective equipment, exclusion, psychological problems, fear, a marginalized personal and family life, a lack of a positive organizational culture, problems related to patients and relationships with their families; to cope with these emerging conditions and overcome these challenges, they used strategies such as performance reliabilities. As a result, these challenges can be mitigated by providing adequate protective equipment, a suitable work environment, more social and financial support, proper shift management, increased attention to nurses’ physical and mental health, and consideration of appropriate mechanisms for nurses’ communication with their families and the families of patients.

## Data Availability

The datasets used and/or analyzed during the current study are available from the corresponding author on reasonable request.

## Abbreviation

PPE: Personal Protective Equipment
